# Fibrodysplasia Ossificans Progressiva: Clinicoradiological Insights Into a Rare Case

**DOI:** 10.7759/cureus.111567

**Published:** 2026-06-26

**Authors:** Gudly Nanda, Biswajit Mohanty, Sugyani Mohapatra, Mamata Singh

**Affiliations:** 1 Neuroimaging and Interventional Radiology, National Institute of Mental Health and Neurosciences, Bangalore, IND; 2 General Medicine, Srirama Chandra Bhanja Medical College Hospital, Cuttack, IND; 3 Radiodiagnosis, Srirama Chandra Bhanja Medical College Hospital, Cuttack, IND; 4 Radiology, Maharaja Jajati Keshari Medical College and Hospital, Jajpur, IND

**Keywords:** acvr1 gene mutation, congenital hallux valgus, fibrodysplasia ossificans progressiva (fop), heterotopic ossification (ho), paraspinal ossification

## Abstract

Fibrodysplasia ossificans progressiva (FOP) is a rare genetic disorder characterised by progressive heterotopic ossification of soft tissues due to mutations in the ACVR1 gene, leading to dysregulated bone morphogenetic protein signalling. Over time, recurrent episodes of painful inflammatory soft tissue swellings (flare-ups) lead to progressive ossification and eventual ankylosis of joints, resulting in severe restriction of mobility. Most patients become wheelchair-bound by the third decade and may succumb to complications such as thoracic insufficiency syndrome and cardiorespiratory compromise. Due to its rarity and lack of awareness, FOP is often misdiagnosed, and patients may undergo unnecessary invasive procedures such as biopsies or surgical excisions, which can precipitate rapid disease progression. Early recognition based on clinical and radiological findings is therefore critical to prevent iatrogenic harm. We present a rare case of a six-year-old girl with this condition, emphasising the diagnostic challenges and key imaging findings.

## Introduction

Fibrodysplasia ossificans progressiva (FOP) is an ultra-rare genetic disorder characterised by progressive heterotopic ossification of soft tissues due to activating mutations in the ACVR1 gene, resulting in dysregulated bone morphogenetic protein (BMP) signalling [1]. This disease is characterised by congenital malformations of the great toes, typically presenting as shortened, monophalangeal, or valgus-deformed halluces. The disease is marked by progressive heterotopic ossification, wherein soft connective tissues such as muscles, tendons, ligaments, fascia, and aponeuroses are gradually replaced by mature bone. Recurrent episodes of painful inflammatory swellings (flare-ups) result in cumulative extraskeletal bone formation, leading to progressive joint ankylosis, severe restriction of mobility, skeletal deformities, and ultimately profound functional disability. Because of its rarity and early resemblance to other soft tissue disorders, FOP is often misdiagnosed, and invasive procedures may accelerate disease progression. Recognition of characteristic clinical and radiological features is therefore crucial for early diagnosis and prevention of iatrogenic harm. We report a rare case of a six-year-old girl with classic clinical and radiological manifestations of FOP.

## Case presentation

**Figure 1:**
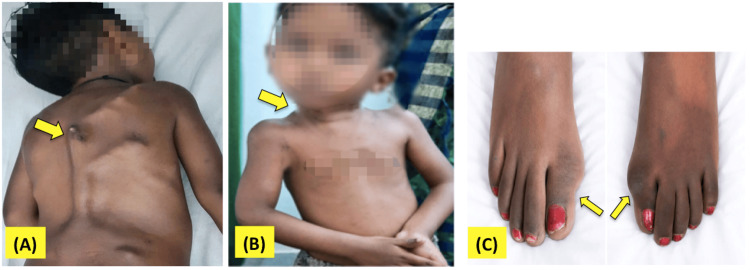
Characteristic phenotypic manifestations of fibrodysplasia ossificans progressiva. (A) Hard nodular prominence over the bilateral paraspinal/scapular region with hard sheet-like midline mass (arrow) corresponding to heterotopic ossification. (B) Torticollis with left-sided neck tilt and restricted mobility of the shoulders. (C) Bilateral congenital hallux valgus deformity, a characteristic hallmark of FOP. FOP: Fibrodysplasia ossificans progressiva

A six-year-old female child presented with progressive complaints of a hard, sheet-like swelling over the midline of the back, restricted movement of the left upper limb, and deviation of the head and neck towards the left side. According to the patient’s parents, the neck deformity was noted shortly after birth and gradually progressed with the development of multiple nodular hard swellings over time. The clinical course was insidious and progressive, leading to increasing functional limitation. There was no significant antenatal or natal history. The patient had no prior history of trauma, infections, or surgical interventions. Family history was unremarkable. Physical examination revealed a sheet-like, non-tender, hard mass overlying the midline of the back (Figure 1A). The lesion had an inverted Y-shaped configuration with branching extensions at the level of the iliac region and interspersed nodularity. Head-to-toe examination also revealed torticollis with deviation of the head to the left (Figure 1B), restricted range of motion of the spine, reduced shoulder mobility, which was more pronounced on the left side, and bilateral hallux valgus deformity (Figure 1C). No focal neurological deficits were observed. Other systemic examinations were normal.

Laboratory investigations, including serum calcium, vitamin D, and parathyroid hormone levels, were within normal limits. Genetic analysis could not be performed due to financial limitations.

**Figure 2:**
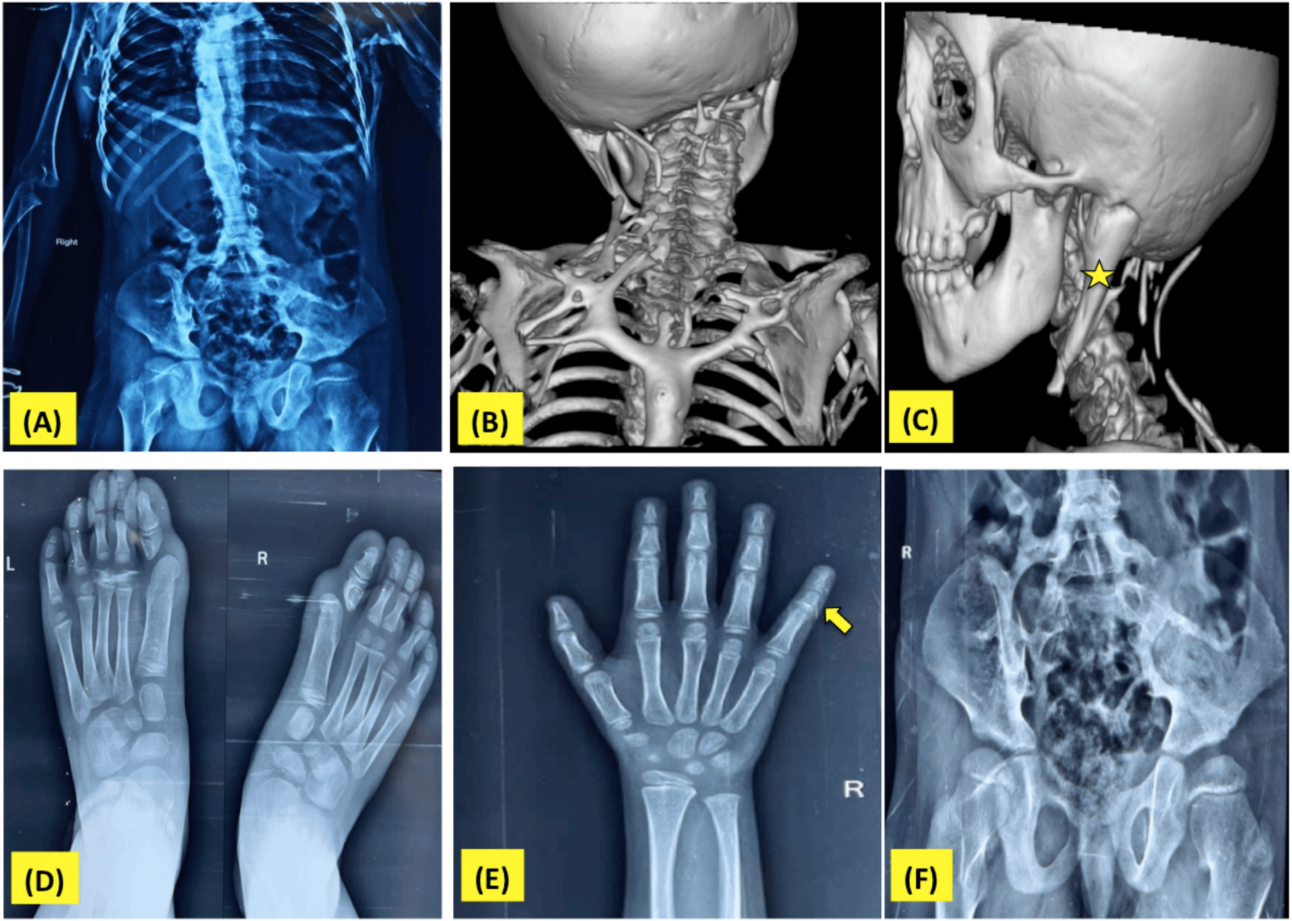
Radiological imaging demonstrating characteristic heterotopic ossification and skeletal anomalies in FOP. (A) X-ray showing a dense sheet-like midline heterotopic ossified band with centrifugal bridging ossifications extending laterally, with pseudo-joint formation with adjacent ribs and iliac bones. (B) VRT image demonstrating extensive heterotopic ossification involving the posterior cervical and upper thoracic paraspinal soft tissues with bridging ossified bands extending towards the scapula. (C) VRT image demonstrating heterotopic ossification along the posterior cervical soft tissues with partial ossification involving the sternocleidomastoid muscle (asterisk), contributing to torticollis and restricted neck movement. (D) Bilateral feet radiographs showing characteristic congenital hallux valgus deformity. (E) Radiograph of the right hand demonstrating shortening of the middle phalanx of the fifth digit (brachymesophalangy) (arrow). (F) Pelvic radiograph revealing broad, short femoral necks bilaterally. FOP: Fibrodysplasia ossificans progressiva; VRT: volume rendering technique

The child underwent comprehensive radiographic and sonographic evaluation. The preliminary radiographs and a subsequent non-contrast CT revealed a dense, sheet-like ossified subcutaneous mass in the midline extending longitudinally overlying the thoracic and upper lumbar spine with interspersed nodularity and focal attachments to the spinous processes at multiple vertebral levels, suggesting progressive incorporation into the axial skeleton. Multiple branching linear ossification bands were seen radiating centrifugally from the midline mass, forming pseudo-articulations with adjacent ribs and iliac blades. There was extensive heterotopic ossification seen within the inter and intramuscular fascial planes of the paraspinal musculature, including trapezius and erector spinae, with bridging ossification extending towards the scapula, forming pseudo-articulations (Figures 2A, 2B). In bilateral shoulder girdle region, ossified bands bridging the scapula (Figure 2B) and surrounding soft tissues were seen, contributing to restriction of shoulder mobility. Additionally, partial ossification of the left sternocleidomastoid muscle (Figure 2C) was also identified, which explained the torticollis. Other associated skeletal anomalies were evident, including bilateral hallux valgus deformity (Figure 2D), shortening of the middle phalanx of the fifth digit (brachymesophalangy) (Figure 2E), and broad femoral necks (Figure 2F). Overall, the radiological findings demonstrated extensive, mature heterotopic ossification in a characteristic axial-to-appendicular distribution, with bridging ossifications and pseudo-joint formation, consistent with advanced FOP.

After the diagnosis, parents were counselled regarding the progressive nature of the disease and the need to avoid trauma and unnecessary invasive procedures that could trigger further heterotopic ossification. The child was subsequently referred to a rehabilitation centre for supportive multidisciplinary care and long-term follow-up.

## Discussion

FOP, also known as Stoneman disease or Münchmeyer disease, is a rare disorder characterised by progressive formation of bone within soft tissues, resulting in increasing restriction of movement and disability. The condition affects approximately one in two million individuals worldwide [1]. The disorder is classically defined by the coexistence of congenital malformations of the great toes and progressive heterotopic ossification in characteristic anatomical patterns [1,2]. The present case fulfils this clinicoradiological pattern: the child had bilateral hallux valgus, progressive axial and paraspinal ossification, restriction of shoulder and spinal mobility, torticollis due to sternocleidomastoid involvement, and associated skeletal anomalies including broad femoral necks and brachymesophalangy.

The molecular basis of classic FOP is an activating mutation in ACVR1/ALK2, a BMP type I receptor, most commonly the recurrent c.617G>A mutation resulting in the p.R206H substitution [1,3]. This gain-of-function alteration leads to dysregulated BMP signalling and aberrant activation of osteogenic pathways. As a result, inflammatory soft tissue lesions undergo a sequence of fibroproliferation, chondrogenesis, and endochondral ossification, ultimately forming mature lamellar heterotopic bone [1,4]. 

The distribution of heterotopic ossification in this child mirrors the characteristic temporal and anatomical progression of FOP. Ossification typically begins in the dorsal, axial, cranial, and proximal regions before progressing toward ventral, appendicular, caudal, and distal sites [1,4]. Early involvement of the neck and paraspinal region is therefore typical and explains the torticollis and restricted spinal mobility seen in our patient. The dense sheet-like ossification over the posterior midline, with branching bands extending toward the ribs, scapula, humerus, and iliac bones, is in keeping with the described formation of ribbons, sheets, and plates of heterotopic bone. The pseudo-articulations or false joints formed between heterotopic bone and adjacent skeletal structures are a recognised radiographic manifestation of advanced FOP and are particularly common around the shoulder girdle and axial skeleton [5,6].

Radiology is central to the diagnosis and safe management of FOP. Plain radiographs are often sufficient to demonstrate congenital great toe malformations, shortened digital elements, broad femoral necks, and mature heterotopic ossification [5,6]. Computed tomography further delineates the three-dimensional extent of ossification, its relationship with the axial skeleton, and the presence of bony bridges or pseudo-articulations, as seen in this case. CT is particularly useful when the ossification is extensive, sheet-like, or anatomically complex, because it can show attachments to spinous processes, ribs, scapulae, iliac bones, and periarticular regions with greater clarity than radiographs [7]. MRI is not routinely required for established mature lesions, but it is useful during acute flare-ups because active preosseous lesions may show muscle enlargement, edema, and inflammatory signal changes before mineralisation becomes apparent [8]. In the present child, sonography demonstrated hyperechoic bony structures in the subcutaneous plane, while radiography and CT provided the decisive diagnostic information.

The major diagnostic pitfall in FOP is misinterpretation of early soft tissue swellings as aggressive juvenile fibromatosis, soft tissue sarcoma, lymphedema, dermatomyositis-related calcinosis, tumoral calcinosis, or nonspecific myositis ossificans [1,7]. Progressive osseous heteroplasia may also enter the differential diagnosis; however, it usually begins with dermal or subcutaneous ossification and lacks the classic congenital great toe phenotype and stereotyped axial-to-appendicular progression of FOP. Traumatic myositis ossificans is typically localised and follows a history of trauma, whereas FOP is multifocal, progressive, and associated with developmental skeletal anomalies. At present, no formal diagnostic criteria have been established for FOP. The diagnosis is largely based on characteristic clinical and imaging findings and may be confirmed by molecular genetic testing for pathogenic variants in the ACVR1 gene. Recognition of malformed great toes is therefore critical. Failure to identify this clue can lead to biopsy or surgical excision, both of which may provoke severe new ossification and worsen disability [1,2]. In our case, the absence of prior biopsy or surgery was important, as iatrogenic trauma can accelerate disease progression. A limitation of this report is the lack of molecular confirmation by ACVR1 gene mutation analysis. Genetic testing could not be performed due to financial constraints and the patient’s subsequent loss to follow-up. However, the characteristic clinical and radiological features that are considered highly suggestive of FOP supported the diagnosis.

The functional burden of FOP is cumulative. The progressive accumulation of heterotopic ossification in FOP leads to increasing functional impairment and disability over time. Ankylosis of major joints and restriction of chest wall mobility can significantly compromise daily activities and quality of life, while thoracic involvement may predispose affected individuals to respiratory complications, a major contributor to long-term morbidity and mortality [1,9]. Recent systematic review data also emphasise that cardiovascular, skeletal, and respiratory comorbidities contribute substantially to morbidity and mortality in FOP [9]. These complications underscore why early recognition is not merely diagnostic but preventive: once mature heterotopic bone develops, it is permanent and surgical removal is generally counterproductive.

Current management is therefore centred on prevention of flare-ups, avoidance of iatrogenic harm, symptomatic treatment, and preservation of function. Patients should avoid intramuscular injections, unnecessary surgical procedures, forceful stretching, contact sports, and other activities likely to cause soft tissue trauma. Dental care should emphasise prevention, and mandibular blocks should be avoided when possible. During acute flare-ups, short courses of corticosteroids may be considered when major joints are threatened, while nonsteroidal anti-inflammatory drugs, analgesics, leukotriene inhibitors, and mast-cell stabilisers may be used for symptomatic relief, although none reliably reverses established disease [1]. Physiotherapy should be gentle and functional rather than aggressive, because forceful range-of-motion exercises may worsen local trauma. Genetic counselling, family education, respiratory surveillance, fall prevention, and long-term multidisciplinary follow-up are essential components of care.

Palovarotene, a retinoic acid receptor γ (RARγ) agonist, is the first FDA-approved drug for reduction in the volume of new heterotopic ossification in eligible patients with FOP: female children aged eight years and older and male children aged 10 years and older. It is a RARγ agonist that inhibits BMP signalling pathways; hence, hindering chondrogenesis and, eventually, heterotopic ossification. The FDA-approved regimen includes chronic daily dosing with flare-up dose escalation in appropriate patients, but it carries important safety warnings, including embryo-fetal toxicity and premature epiphyseal closure in growing pediatric patients. Therefore, its use requires specialist supervision, careful age and skeletal maturity assessment, and individualised risk-benefit consideration [10]. Considering the age of the patient in our case, Palovarotene was not considered a suitable treatment option, as current FDA approval for its use is restricted to female children aged ≥8 years. Therefore, its administration was not applicable in the present case.

This case is notable because it demonstrates several classic and radiologically striking features of FOP in early childhood: congenital hallux valgus, broad femoral necks, fifth-finger brachymesophalangy, extensive sheet-like paraspinal ossification, branching ossified bands, sternocleidomastoid involvement causing torticollis, and multiple pseudo-articulations involving the shoulder girdle and axial skeleton. The case highlights the decisive role of imaging in establishing the diagnosis without biopsy and reinforces the need for awareness among radiologists, paediatricians, orthopedicians, and surgeons. In any child presenting with progressive hard soft tissue masses, restricted mobility, and malformed great toes, FOP should be considered early so that harmful invasive procedures can be avoided, and appropriate counselling can be initiated.

## Conclusions

FOP is a rare but highly disabling disorder that can be reliably diagnosed based on characteristic clinical features, particularly congenital hallux valgus, and distinctive imaging findings. Early recognition is essential to avoid iatrogenic harm and to enable appropriate multidisciplinary management of this progressively disabling disorder. Recent advances, including the development of Palovarotene, offer promising therapeutic options; however, prevention of trauma and careful patient management remain the cornerstone of care. Increased awareness among healthcare professionals is vital to improve outcomes, reduce morbidity, and ensure appropriate counselling and support for affected patients and their families.
